# Selective Biosynthesis of Furoic Acid From Furfural by *Pseudomonas Putida* and Identification of Molybdate Transporter Involvement in Furfural Oxidation

**DOI:** 10.3389/fchem.2020.587456

**Published:** 2020-09-25

**Authors:** Zhaojuan Zheng, Qianqian Xu, Huanghong Tan, Feng Zhou, Jia Ouyang

**Affiliations:** ^1^Jiangsu Province Key Laboratory of Green Biomass-Based Fuels and Chemicals, Jiangsu Co-Innovation Center of Efficient Processing and Utilization of Forest Resources, College of Chemical Engineering, Nanjing Forestry University, Nanjing, China; ^2^Key Laboratory of Forestry Genetics and Biotechnology of Ministry of Education, Nanjing Forestry University, Nanjing, China

**Keywords:** biocatalysis, furoic acid, *Pseudomonas putida*, selective oxidation, molybdate transporter

## Abstract

Upgrading of furanic aldehydes to their corresponding furancarboxylic acids has received considerable interest recently. Herein we reported selective oxidation of furfural (FAL) to furoic acid (FA) with quantitative yield using whole-cells of *Pseudomonas putida* KT2440. The biocatalytic capacity could be substantially promoted through adding 5-hydroxymethylfurfural into media at the middle exponential growth phase. The reaction pH and cell dosage had notable impacts on both FA titer and selectivity. Based on the validation of key factors for FAL conversion, the capacity of *P. putida* KT2440 to produce FAL was substantially improved. In batch bioconversion, 170 mM FA was produced with selectivity nearly 100% in 2 h, whereas 204 mM FA was produced with selectivity above 97% in 3 h in fed-batch bioconversion. Particularly, the role of molybdate transporter in oxidation of FAL and 5-hydroxymethylfurfural was demonstrated for the first time. The furancarboxylic acids synthesis was repressed markedly by destroying molybdate transporter, which implied Mo-dependent enzyme/molybdoenzyme played pivotal role in such oxidation reactions. This research further highlights the potential of *P. putida* KT2440 as next generation industrial workhorse and provides a novel understanding of molybdoenzyme in oxidation of furanic aldehydes.

**Graphical Abstract:**
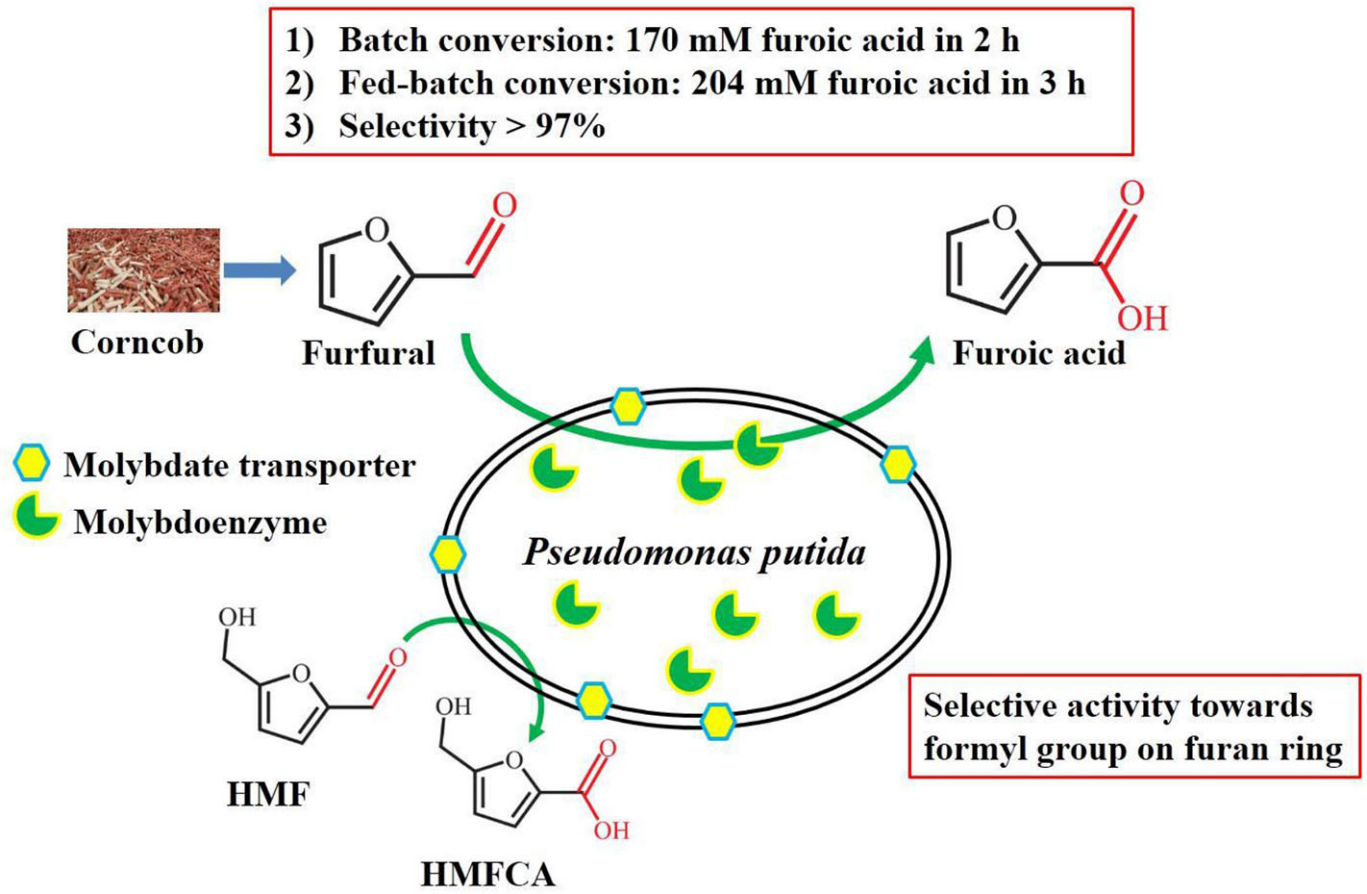
Reaction accomplished in this study.

## Introduction

Lignocellulosic biomass is the most abundant and sustainable resource from which many kinds of platform chemicals can be derived (Den et al., 2018). 5-hydroxymethylfurfural (HMF) is accessible from glucose and cellulose while furfural (FAL) derives from xylose and C_5_-rich hemicellulose in the presence of acid catalysts (Mika et al., 2018). Both FAL and HMF are versatile platform molecules that can be converted into a variety of important chemicals, due to the presence of active groups such as primary hydroxyl and formyl (Mariscal et al., 2016; Hu et al., 2018). Selective oxidation of FAL and HMF to a variety of highly functionalized carboxylic acids and hydroxyl acids was an important aspect in their upgrading (Mariscal et al., 2016; Hu et al., 2018). FAL can be oxidized to furoic acid (FA) (Mariscal et al., 2016; Shi et al., 2019; Wang et al., 2020), while HMF to 5-hydroxymethylfuroic acid (HMFCA), 2,5-diformylfuran, 5-formylfuroic acid, and 2,5-furandicarboxylic acid (FDCA) (Qin et al., 2015; Hu et al., 2018). Up to now, chemocatalytic approaches for selective oxidation of FAL and HMF have been extensively studied, which often involve the use of toxic and hazardous reagents and solvents, the harsh conditions, as well as the low selectivity (Mariscal et al., 2016; Sajid et al., 2018). In the case of FA, it is currently produced from FAL industrially *via* a Cannizzaro reaction with NaOH, accompanied by the byproduct furfuryl alcohol (FOL) formation, resulting in a very low selectivity (Mariscal et al., 2016).

An attractive alternative exists in the biocatalytic transformation that performs reactions with much higher selectivity under ambient conditions. Efforts with regards to biocatalysis have been made at achieving selective oxidation of FAL to FA. Based on catalytic promiscuity of enzymes, recombinant *Escherichia coli* strains harboring various heterogeneous enzymes were reported for FA production. These enzymes included 3-succinoylsemialdehyde-pyridine dehydrogenase (SAPDH) (Shi et al., 2019), horse liver alcohol dehydrogenase (Peng et al., 2019), and HMF oxidase (HMFO) variant (Wang et al., 2020). Likewise, microbes resistant to FAL were also isolated and implemented for FA production. *Nocardia corallina* B-276 oxidized FAL of 9 g/L to FA with yield of 92% using resting cells (Pérez et al., 2009). *Gluconobacter oxydans* ATCC 621H converted FAL into FA with concentration exceeding 40 g/L and yield close to 100% using a compressed oxygen supply-sealed and stirred tank reactor (Zhou et al., 2017). In view of their good performances, it could be envisioned that these bacteria harbored relevant enzymes with high activities toward FAL and other furanic aldehydes. However, researches focusing on identification of such enzymes were limited except for few reports (Koopman et al., 2010; Wang X. et al., 2015).

The trace element molybdenum is essential in the prokaryotic and eukaryotic organisms, and it participates in the biosynthesis of molybdenum cofactor that forms the catalytic center of a vast variety of molybdoenzymes (Schwarz et al., 2009). In nature, molybdenum is bioavailable in the form of molybdate (${\text{MoO}}_{4}^{2-}$), which is transported by an ABC-type transporter comprising three proteins, ModA (molybdate binding protein), ModB (membrane protein) and ModC (the ATPase) (Self et al., 2001). Once entering into the cell, molybdate is subsequently incorporated into the complex pathway of molybdenum cofactor biosynthesis and finally to activate molybdoenzymes (Hille et al., 2014; Mendel and Leimkühler, 2015; Leimkühler and Iobbi-Nivol, 2016). Researches had showed that ModABC played a critical role in the detoxification of aromatic compounds, such as nicotine and quinoline (Blaschke et al., 1991; Ganas et al., 2008; Xia et al., 2018). Moreover, molybdate uptake was also involved in aerobic degradation of FA, as a 2-furoyl-CoA dehydrogenase which converted 2-furoyl-CoA into 5-hydroxy-2-furoyl-CoA was confirmed as molybdoenzyme (Koenig and Andreesen, 1989, 1990; Koopman et al., 2010). Despite the importance of molybdate transporter in metabolism of aromatic compounds, there has been no work focusing on its functionality in biotransformation of furanic aldehydes to furancarboxylic acids.

As well-known, bacteria from the genus *Pseudomonas* exhibit an intrinsic resistance to a variety of toxic compounds. Among them, the *Pseudomonas putida* KT2440 has a profound impact on biotechnology through its use in the degradation of aromatic compounds and production of value-added products (Jiménez et al., 2002; Belda et al., 2016). In this paper, the application potential of *P. putida* KT2440 was developed as outstanding FA producer. The catalytic performance of this strain was substantially enhanced upon HMF adaption and process optimization. Moreover, it was for the first time to uncover that a molybdate transporter (PP_3828–PP_3830) played an indispensable role in oxidation of formyl groups of FAL and other furanic aldehydes, which would guide us to discover unknown molybdoenzyme as novel biocatalyst for oxidative upgrading of bio-based furanic aldehydes.

## Materials and Methods

### Materials

FAL, FA, and FOL were purchased from Aladdin (Shanghai, China). HMF, HMFCA, and 2,5-bis(hydroxymethyl)furan (BHMF) were purchased from Adamas Reagent Co., Ltd. (Shanghai, China). Yeast extract and tryptone were purchased from Oxoid (Cambridge, UK). Corncob was provided by Jiangsu Kangwei Biologic Co., Ltd. (Dongtai, China). All chemicals were of analytical grade.

### Preparation of Whole-Cell Biocatalyst

Generally, *P. putida* KT2440 was grown in Luria-Bertani (LB) medium at 30°C and 200 rpm for 12 h on a rotary shaker. Then, 1% seed culture was inoculated to the fresh LB medium and cultivated under the same conditions. At the middle exponential growth phase, 4.5 mM HMF was added to the medium to promote the biocatalytic activity of cells. To investigate the effect of the type of inducer, HMF was substituted by FAL and FOL. To study the effect of HMF addition moment, HMF was added at early, middle and end exponential growth phase (based on cell density), respectively. The effect of HMF addition amount was also surveyed, ranging from 1.5 to 8 mM. After incubation for 12 h, cells were harvested by centrifugation with 6,000 *g* for 8 min, washed twice with 0.85% NaCl saline solution, and resuspended in the corresponding buffer for subsequent experiments.

### Preparation of FAL From Corncob

The corncob was air-dried and ground to particle size 10–20 mesh. Depolymerization of corncob and dehydration of therein xylose to FAL was conducted in a 30 mL sealed stainless steel reactor, which contained a mixture of 3 g corncob and 24 mL of 2% (w/w) H_2_SO_4_ at a solid-to-liquid ratio of 1:8. The reaction mixture was rapidly heated to 160°C in oil bath and held for 100 min without shaken. After that, the reaction vessel was quickly cooled with cold water to room temperature. Crude FAL was obtained from the reaction mixture by liquid/liquid extraction of dichloromethane, followed by solvent evaporation at 80°C.

### Whole-Cell Catalytic Oxidation of FAL to FA

Generally, 20 mL of phosphate buffer (200 mM, pH 6.0) containing 50 mM HMF, 10.5 g/L cells (dry cell weight), and 25 mM CaCO_3_ was incubated at 30°C, pH 6.0, and 200 rpm. The effect of pH was studied by performing the reaction in the buffer with pH adjusted to 5.5, 6.0, 6.5, 7.0, and 7.5, respectively. The influence of cell dosage was measured ranging from 5.25 g/L to 12.25 g/L cells (dry cell weight) at intervals of 1.75 g/L. To evaluate the tolerance of *P. putida* KT2440 toward FAL, the FAL concentration was designated in the range of 20–175 mM, and CaCO_3_ concentration was adjusted upon FAL, at 1/2 of the added FAL by molar. Aliquots were withdrawn from the reaction mixtures at specified times and diluted with the corresponding mobile phase prior to HPLC analysis.

For fed-batch conversion, 50 mL of phosphate buffer (200 mM, pH 6.0) containing 65 mM FAL, 10.5 g/L cells (dry cell weight), and 50 mM CaCO_3_ was incubated at 30°C, pH 6.0, and 200 rpm. During biotransformation, once the FAL was nearly depleted, FAL was repeatedly fed into the reaction mixture for thrice, and samples were collected for monitoring its conversion.

### Construct of *modA* Disruption Mutant

The construction of a *modA* disruption mutant was performed using a homologous recombination gene replacement system. All procedures were conducted according to a previous reference with minor modification (Wang Y. et al., 2015). Briefly, genomic DNA of *P. putida* KT2440 was extracted through the Wizard Genomic DNA Purification Kit (Promega, Madison, WI, USA). The flanking regions of *modA* gene were amplified from *P. putida* KT2440 genomic DNA using the primers *modA*up.f and *modA*up.r (upstream, ~500 bp) and *modA*down.f and *modA*down.r (downstream, ~500 bp), respectively (Supplementary Table 1). After gel purification, overlap extension PCR was performed, wherein the upstream and downstream regions were fused using the primers *modA*up.f and *modA*down.r. The resulting PCR product was gel purified and ligated into the suicide vector pK18*mobsacB* to form a new plasmid pK18MS-Δ*modA*. Plasmid pK18MS-Δ*modA* was transformed into *P. putida* KT2440 by electroporation. Single crossover recombinants containing the integration of plasmid pK18MS-Δ*modA* into the chromosome of *P. putida* KT2440 were obtained by plating the cells on LB agar containing 50 mg/L kanamycin. After two rounds of propagation in LB broth containing 15% (w/v) sucrose but without antibiotic, the double-crossover recombinants were screened by culture on LB agar containing 15% (w/v) sucrose. All the constructed strains were validated by PCR and DNA sequencing.

### Analytical Methods

The concentrations of FAL, FA, FOL, HMF, HMFCA, and BHMF in the reaction mixture were determined by an HPLC system (Agilent 1260 series, USA) equipped with an Aminex HPX-87H column (300 × 7.8 mm, Bio-Rad, USA) and a refractive index detector (Shimadzu, Japan) at 65°C. The column was eluted with 5 mM H_2_SO_4_ at a flow rate of 0.6 mL/min and 55°C.

The initial reaction rate (V_0_) was calculated by the increased concentration of FA during the initial reaction stage of 10 min. The conversion was defined as the ratio of the consumed FAL amount to the initial FAL amount (in mol). The yield was defined as the ratio of the formed FA amount to the theoretical value based on the initial FAL amount (in mol). The selectivity was defined as the ratio of the formed FA amount to the total amount of all products (in mol). The reaction productivity was calculated using the following equation

$$\begin{array}{l} \text{Productivity}\left(\text{mM}/\text{h}\right)=\frac{dp\left(\text{mM}\right)}{dt\;\left(\text{h}\right)} \end{array}$$

where (*p*) is the product and (*t*) is the time. All the experiments were conducted at least in duplicate, and the values were expressed as mean ± standard deviations.

## Results and Discussion

### Addition of FAL and FAL Analogs to Boost the Catalytic Performance of Whole-Cell Biocatalyst

**Figure 1:**
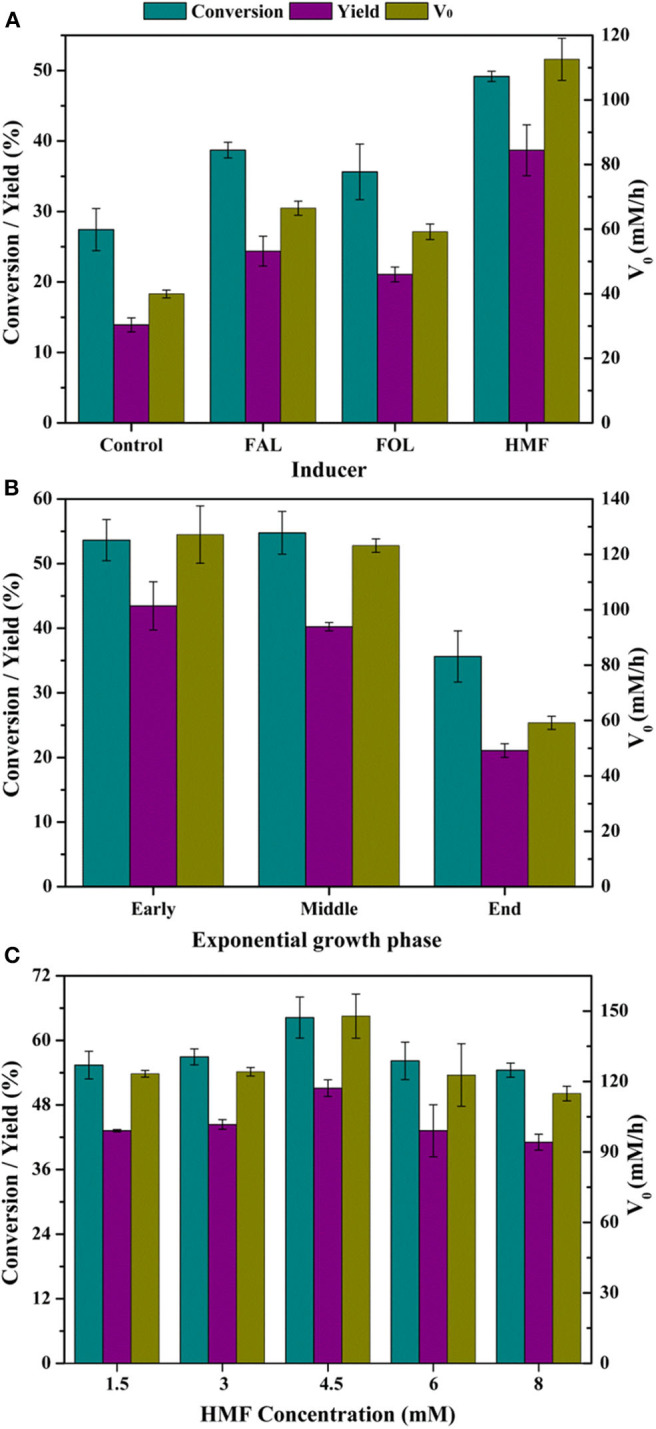
Effect of induction on catalytic performances of *P. putida* KT2440. Reaction conditions: phosphate buffer (200 mM, pH 7.0), 50 mM FAL, 7 g/L cells (dry weight), CaCO_3_ at half molar concentration of FAL, 30°C, 200 rpm, 10 min. **(A)** Various inducers were added with 3 mM at the early exponential growth phase; **(B)** 3 mM HMF was added at different growth phases as inducer; **(C)** HMF was added with designated concentrations at the middle exponential growth phase.

Efficient whole-cell biocatalysts are critical for economically feasible biocatalysis process. Addition of substrate or substrate analogs at very low concentration to the medium had been proved as an effective approach to enhance the bioconversion performance of biocatalyst (Wen et al., 2020). The additives might induce the expression of certain enzyme(s) responsible for substrate transformation. Therefore, to boost the activity of FAL oxidation in *P. putida* KT2440, FOL, FAL, and HMF were added during cultivation and the bioconversion abilities of resultant whole-cells were determined. The results showed all three kinds of furan additives exerted positive influence on bioconversion, since all tested parameters obtained from adapted cells were superior to that from the control (Figure 1A). Therein, HMF exhibited the best positive effect on conversion, yield, and V_0_, followed by FAL and then FOL. The conversion was significantly improved from 27.4 to 49.2%, the yield from 13.9 to 38.7%, and the V_0_ from 39.9 to 112.6 mM/h. It should be pointed out that all aforementioned data were calculated on the basis of samples withdrawn at 10 min, and given enough time, FAL would be completely oxidized to FA in all cases. Besides, it was worth noting that the cell density reduced by ~10% during cultivation with HMF and FOL addition, while decreased by more than 20% in the presence of FAL, which might be due to the most serious toxicity of FAL to cells. Zhang et al. (2019) reported that the FAL-adapted yeast cells showed no improvements in the synthesis of FOL compared to cells free of substrate adaptation. All these results indicated that FAL and FOL might not be an appropriate option as additive into media.

The influence of HMF addition time was shown in Figure 1B, and it was found that addition at early exponential growth phase and middle exponential growth phase was vastly superior to addition at the end exponential growth phase. However, when HMF was added at early exponential growth phase, the final cell density was very low. Therefore, addition at the middle exponential growth phase was favorable to the biotransformation process.

Given these facts, HMF was added at middle exponential growth phase in the range of 1.5–8 mM to promote whole-cell catalytic synthesis of FA. Figure 1C showed that 4.5 mM was the most suitable concentration accompanied by the best catalytic performance of cells, while HMF with both 1.5–3 mM and 6–8 mM gave second stimulation (Figure 1B). Low concentration of HMF with 1.5–3 mM might do not guarantee abundant enzyme activities, whereas high concentration of HMF resulted in inevitable cell damage because of the formation of reactive oxygen species (ROS), causing ROS-associated damage to proteins, nucleic acids, and cell organelles (Wierckx et al., 2011; Zhu et al., 2019). For *P. putida* KT2440, such damage might occur at 6–8 mM HMF, which, in turn, reduced catalytic activity of cells. Nevertheless, compared with control, the cells adapted to HMF with any tested concentration were all conducive to FAL oxidation, since all results in Figure 1C were better than that of control in Figure 1A.

### Optimization of Biotransformation Conditions for FA Production

**Figure 2:**
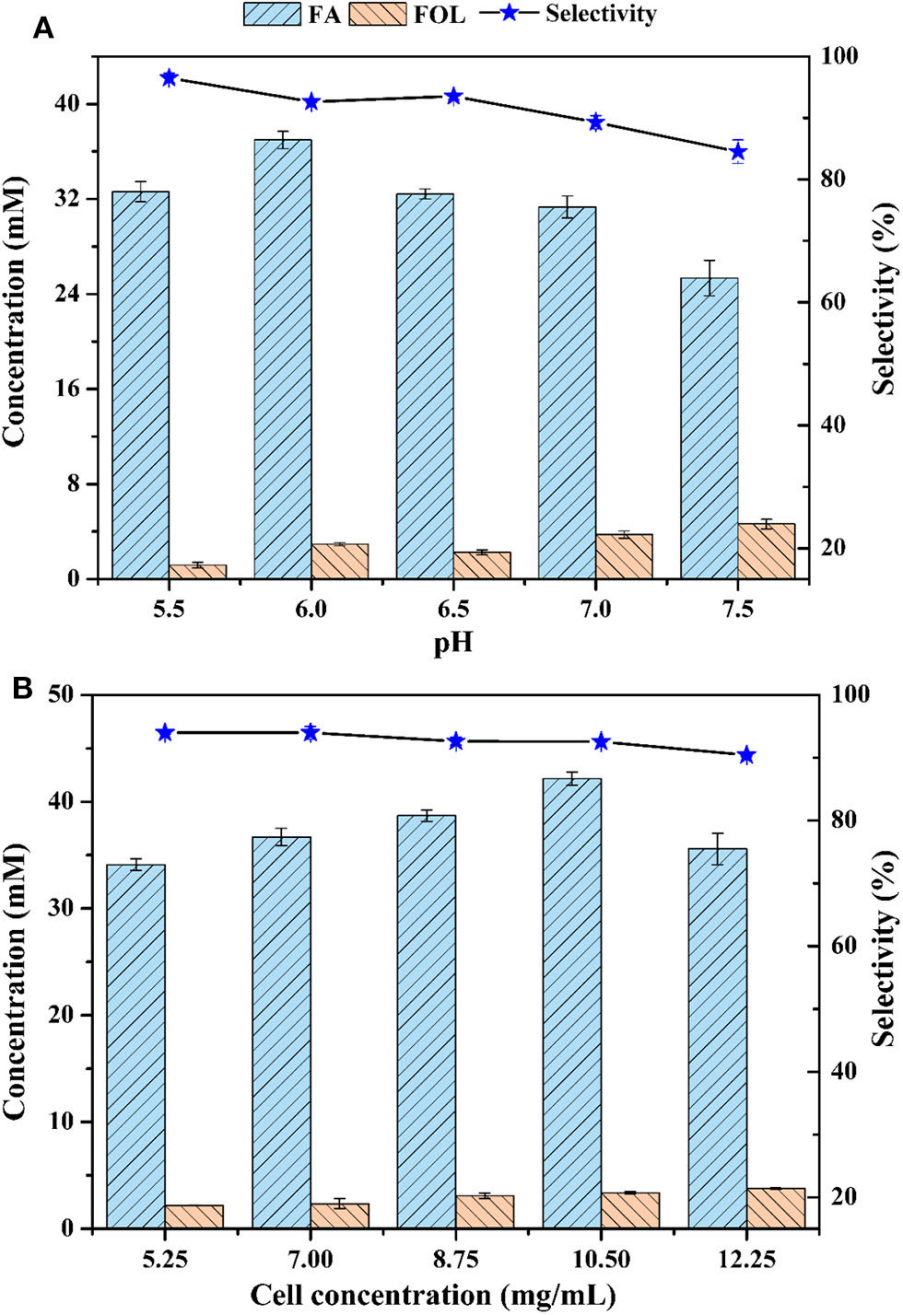
Effect of pH **(A)** and cell concentration **(B)** on biocatalytic oxidation of FAL. Reaction conditions: phosphate buffer (200 mM) with designated pH for **(A)** and pH 6.0 for **(B)**, 70 mM FAL, 7 g/L cells (dry weight) for **(A)** and designated cell concentrations (dry weight) for **(B)**. Other parameters were same as Figure 1.

A major advantage of whole-cells is that cells provide a natural environment for the enzymes, preventing loss of activity in complex reaction systems. However, contrarily to pure enzymes, side-reactions are inevitable for whole-cell biotransformation, which always is the most important issue to be considered (de Carvalho, 2011). In terms of the whole-cell bioconversion of FAL, oxidizing to FA and reducing to FOL usually occurred together in cells, and the concomitant FOL would greatly reduce the selectivity of FA (Wang et al., 2020). Figure 2A showed the effects of reaction pH on FA and FOL production when pH values varied from 5.5 to 7.5. The trend of FA concentration was inverted U-shape curve, whereas the trend of FOL kept rising, likely due to the different optimal pHs of enzymes responsible for oxidation and reduction of FAL. With the increased pH, the maximum titer of FA was raised from 32.6 mM (pH 5.5) to 37.0 mM (pH 6.0) and then back to 25.3 mM (pH 7.5). In terms of selectivity, it dropped from 96.5 to 84.5% with increased pHs. We also found that all FOL would be further oxidized into FA with enough time, assuming the redox reaction between FOL and FAL was reversible, which was consistent with previous reports on *Cupriavidus basilensis* HMF14 and *Amorphotheca resinae* ZN1 (Koopman et al., 2010; Wang X. et al., 2015). However, the accumulated FOL throughout the bioconversion process was much lower for *P. putida* KT2440 than that for *C. basilensis* HMF14 and *A. resinae* ZN1. For the latter two strains, the intermediate FOL could reach half of the starting FAL (Koopman et al., 2010; Wang X. et al., 2015). Based on these, it can be predicted that *P. putida* KT2440 harbors weak enzymatic activity for FAL reduction, which make it an outstanding workhorse for FAL oxidation.

Effects of cell dosage on FAL bioconversion were investigated at pH 6.0. Figure 2B showed that, similar to Figure 2A, the concentration of FA rose first and then fell with the increasing of cell dosage. The highest concentration of 42.2 mM was obtained at cell loading of 10.5 g/L. In contrast, the concentration of FOL kept rising. It was plausible that oxygen became a limiting factor as a result of the enhanced cell biomass and the oxidation reaction toward FAL was, to a large extent, necessary for oxygen although the enzyme(s) involved in this process was uncovered. Unlike the oxidation of FAL, its reduction was often efficiently occurred in facultative anaerobic strains, such as *Bacillus coagulans* and *Saccharomyces cerevisiae* (Yan et al., 2018, 2019), indicating the non-critical role of oxygen. On the whole, the impact of cell dosage on the selectivity was slightly lower than that of pH. The following experiments for FAL bioconversion were conducted with cell dosage of 10.5 g/L.

### Bioconversion of FAL With High Loading Under Optimal Conditions

**Figure 3:**
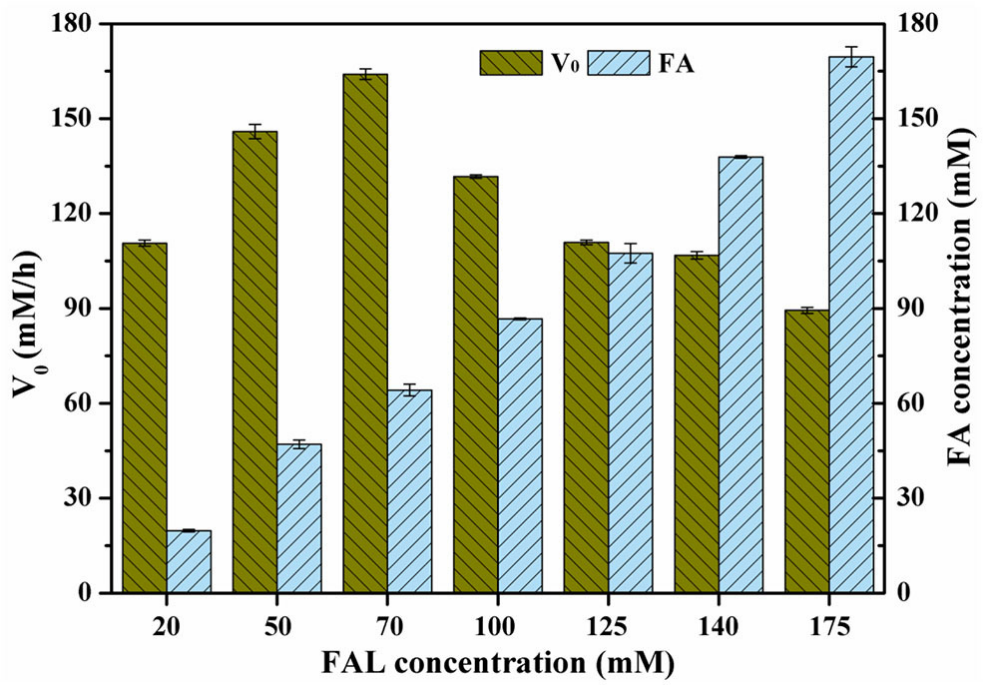
Effect of FAL concentration on FA production. Reaction conditions: phosphate buffer (200 mM, pH 6.0), designated FAL concentrations, 10.5 g/L cells (dry weight), CaCO_3_ at half molar concentration of FAL, 30°C, 200 rpm. The FA concentrations in conversion of 20, 50, 70, 100, and 125 mM FAL were determined on the basis of samples withdrawn at 1 h. The FA concentrations in conversion of 140 and 175 mM FAL were determined on the basis of samples withdrawn at 2 h.

Many studies have focused on the inhibition of furans toward microorganisms (Zaldivar et al., 1999; Franden et al., 2013). In these works, inhibitory activity of furans is to a large extent ascribed to their hydrophobicities. Among them, FAL is generally recognized as the most harmful substance, which might be explained by its strong hydrophobicity (log*P*_FAL_ = 0.41, log*P*_HMF_ = −0.37) (Franden et al., 2013). Therefore, the tolerance of strains to FAL is extremely essential to the high production of FAL-derived chemicals. To evaluate the tolerance of *P. putida* KT2440 toward FAL and its bioconversion ability to produce FA, cells were prepared and exposed to FAL in a wide range of 20–175 mM. Unexpectedly, the final FA concentration was remarkably improved linearly upon the addition amount of FAL, steadily rising to 170 mM with yield of 97.1%. Trace of FOL was formed in the initial reaction period, which was re-oxidized to FA as no FOL could be detected in the end. And as a consequence, the selectivity in all investigations was close to 100% (Figure 3). Given these facts, it could be concluded that the tolerant level of *P. putida* KT2440 was >175 mM under the cultivation and reaction conditions of this study. Of the recently reported strains, each one displayed a distinct tolerant profile. The oxidation of FAL by *E. coli* harboring SAPDH became very sluggish when FAL concentration was improved to 125 mM (Shi et al., 2019; Cheng et al., 2020), whereas the yield of FA sharply decreased to 32.1% using 75 mM FAL as initial substrate in the case of whole-cells of *E. coli* harboring HMFO variant (Wang et al., 2020). The substrate-adapted *Comamonas testosteroni* SC1588 cells could convert 50 mM FAL to the desired product with yield of 96% in 24 h, but only yield of 64% from 100 mM FAL even with the prolonged reaction time of 120 h (Wen et al., 2020). The performance of *G. oxydans* ATCC 621H was comparatively better, which gave satisfactory yield from 104 mM FAL, and the yield decreased by 60% when FAL concentration was increased to 158 mM (Zhou et al., 2017).

Although FAL concentration had no significant effect on the maximal yields (above 95%) and selectivities (nearly 100%), its influence on the reaction rates was substantial. As shown in Figure 3, the highest initial reaction rate of 164.1 mM/h was observed when FAL concentration was 70 mM. Further increase in FAL concentration resulted in a moderately substrate inhibition, since the initial reaction rate dropped to 131.7–89.4 mM/h at the FAL concentrations of 100–175 mM. Anyhow, the initial reaction rate remained satisfactory even at high FAL concentration of 175 mM. As previously described, FAL has more inhibitive effect on microorganisms than HMF, but the V_0_ in this study was considerably high than those for HMF in our previous results (Xu et al., 2020). A reasonable explanation was that the strategy developed in this study to enhance the catalytic performances of cells was valid and reliable, which was also evidenced by the difference between the crude activities of inducer-free cells and HMF-induced cells (Supplementary Figure 1). Compared to the microorganisms reported previously (Zhou et al., 2017; Shi et al., 2019; Cheng et al., 2020; Wang et al., 2020; Wen et al., 2020), biocatalysts of this study appeared to be much more prevailing in synthesis of FA from FAL, because of its high tolerance toward the substrate as well as good catalytic performances.

**Figure 4:**
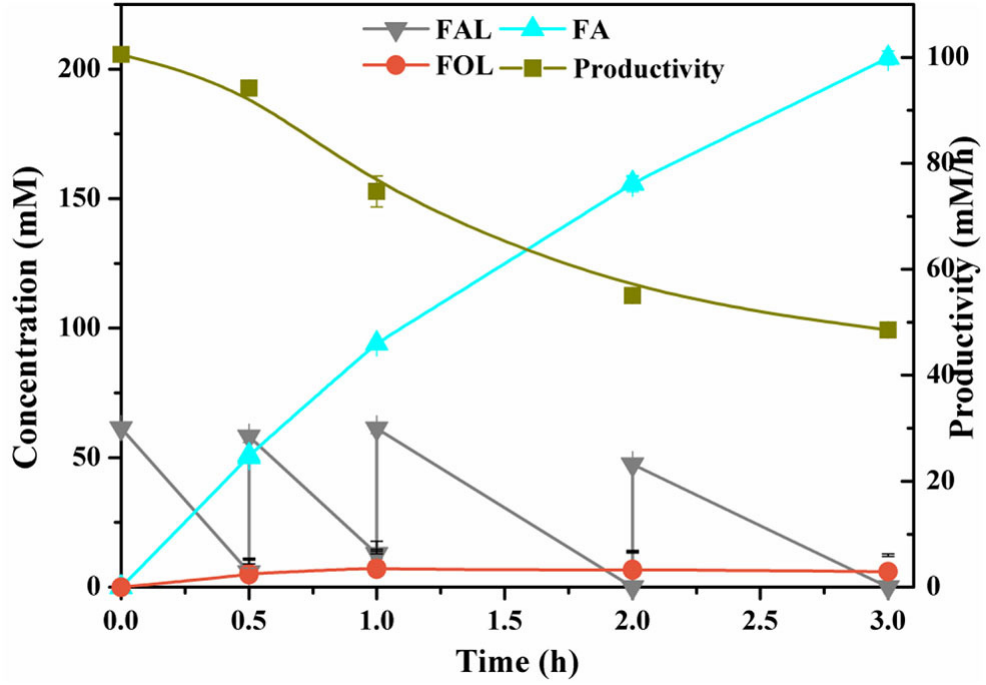
Profiles of selective oxidation of FAL to FA in fed-batch conversion.

High product titers and productivities are eminently desirable in the industrial processes. In view of the inhibition of FAL against enzymes and microorganisms, high loadings of FAL would encounter low biocatalytic efficiency, thus, fed-batch was an effective approach to cope with such inhibition. With the periodical addition of FAL at ~60–70 mM, FA concentration increased rapidly and steadily, demonstrating the strong viability of cells and feasibility of fed-batch strategy. Up to 204 mM FA was produced within 3 h after three-batch feeding of FAL, with total conversion of 100% and yield of 97.5%. The productivities were changed from 100.5 to 48.5 mM/h (Figure 4). The FA produced was not further degraded in contrast to reports on certain strains of genus *Pseudomonas*, which was proved to utilize FA as sole carbon (Wierckx et al., 2011). In addition, only trace of FOL was formed as the sole byproduct and, hence the selectivity of the desired product reached >97% (Figure 4). Guarnieri et al. (2017) cultivated *P. putida* KT2440 in M9 medium supplemented with 1 g/L FAL as sole carbon, and they observed 50% reduction in FAL accompanied by notable accumulation of FOL but no FA after 16 h. This is obvious differently from our results. Generally, FOL accumulation was more commonly occurred in *Saccharomyces cerevisiae, Corynebacterium glutamicum*, etc. under anaerobic conditions (Ishii et al., 2013; Tsuge et al., 2014).

**Figure 5:**
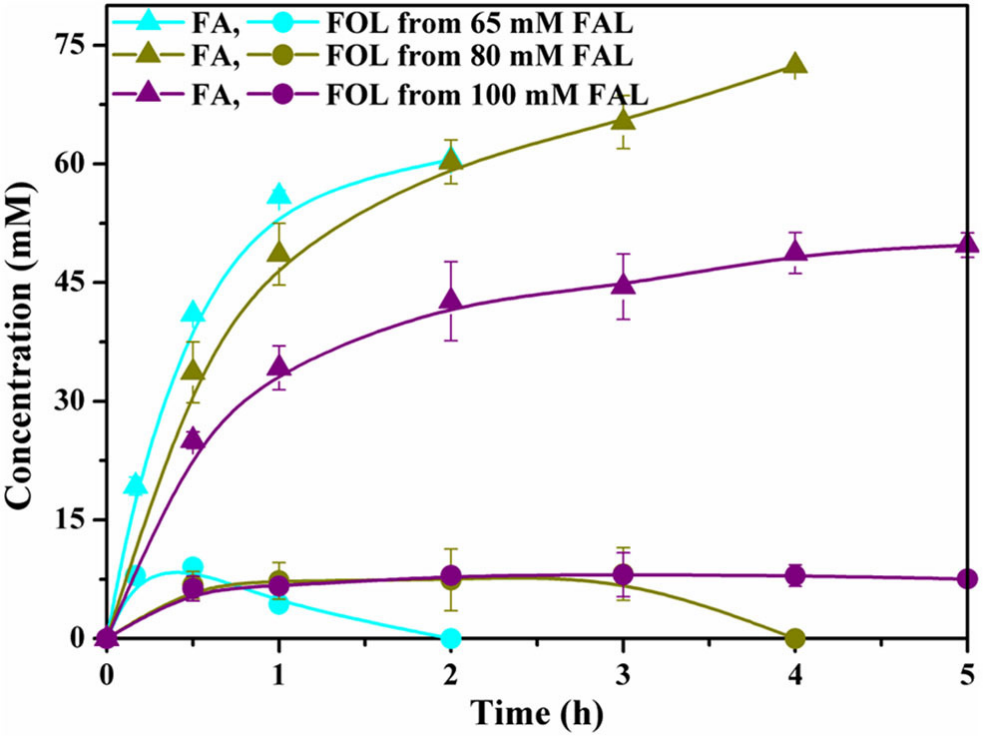
Profiles of selective oxidation of partially purified FAL (Cyan, 65 mM; Dark yellow, 80 mM; Purple, 100 mM) to FA under optimized biotransformation conditions.

We also investigated the potential of cells to biosynthesize of FA from simply prepared and partially purified substrate. In this case, FAL was self-made *via* dilute acid-catalyzed dehydration of corncob followed by dichloromethane extraction. It was found that FAL within 80 mM could be completely depleted with good yields of above 90%. FOL was accumulated in the initial phase of the reaction and re-oxidized finally, leading to FA with selectivity of nearly 100%. We eventually increased the substrate concentration to 100 mM, but under such conditions, both the yield and productivity were profoundly decreased (Figure 5). It might be attributed to the toxic effects of the residual solvent and other undetectable detrimental compounds present in the partially purified FAL on biocatalytic activities of cells, since the crude FAL was directly obtained from corncob through one-step treatment. Currently, except for limited examples, the FAL concentrations used in whole-cell biocatalysis system are usually low or it takes a long time to achieve high yields. Compared with recent reports, the results of this study afforded a promising biocatalytic approach to FA production and further demonstrate the feasibility of biocatalysis in synthesis of biobased furan-derived chemicals (Supplementary Table 2).

### The Role of Molybdate Transporter in Furanic Aldehyde Oxidation

After demonstrating that *P. putida* KT2440 was a good biocatalyst for conversion of FAL and HMF to their corresponding carboxylic acids, we expected to better understand the enzyme involved in such oxidation reactions. Previously, the HMF/furfural oxidoreductase (HmfH) from *C. basilensis* HMF14 and HMFO from *Methylovorus* sp. strain MP688 were proved with activities toward both FAL and HMF, yielding FA and FDCA as products (Koopman et al., 2010; Dijkman and Fraaije, 2014). And the synthetic potential of these biocatalysts in furancarboxylic acids has been fully exploited and developed in recent years (Yuan et al., 2018; Wang et al., 2020). Nevertheless, the target enzyme in our research was likely different from them, as only the formyl group was oxidized by *P. putida* KT2440, evidenced by the conversion of HMF to HMFCA (Xu et al., 2020).

**Figure 6:**
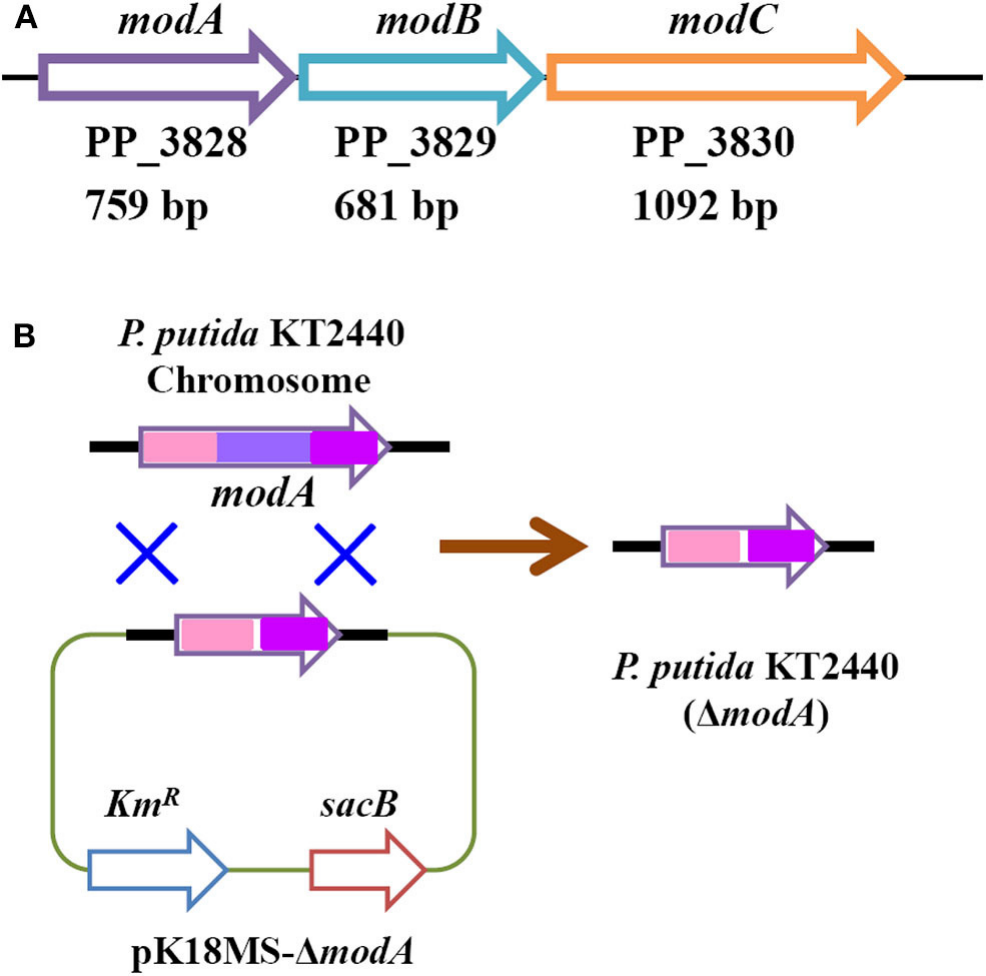
Organization of molybdate transporter operon in *P. putida* KT2440 **(A)** and construction of *modA*-disrupted mutant strain **(B)**.

**Figure 7:**
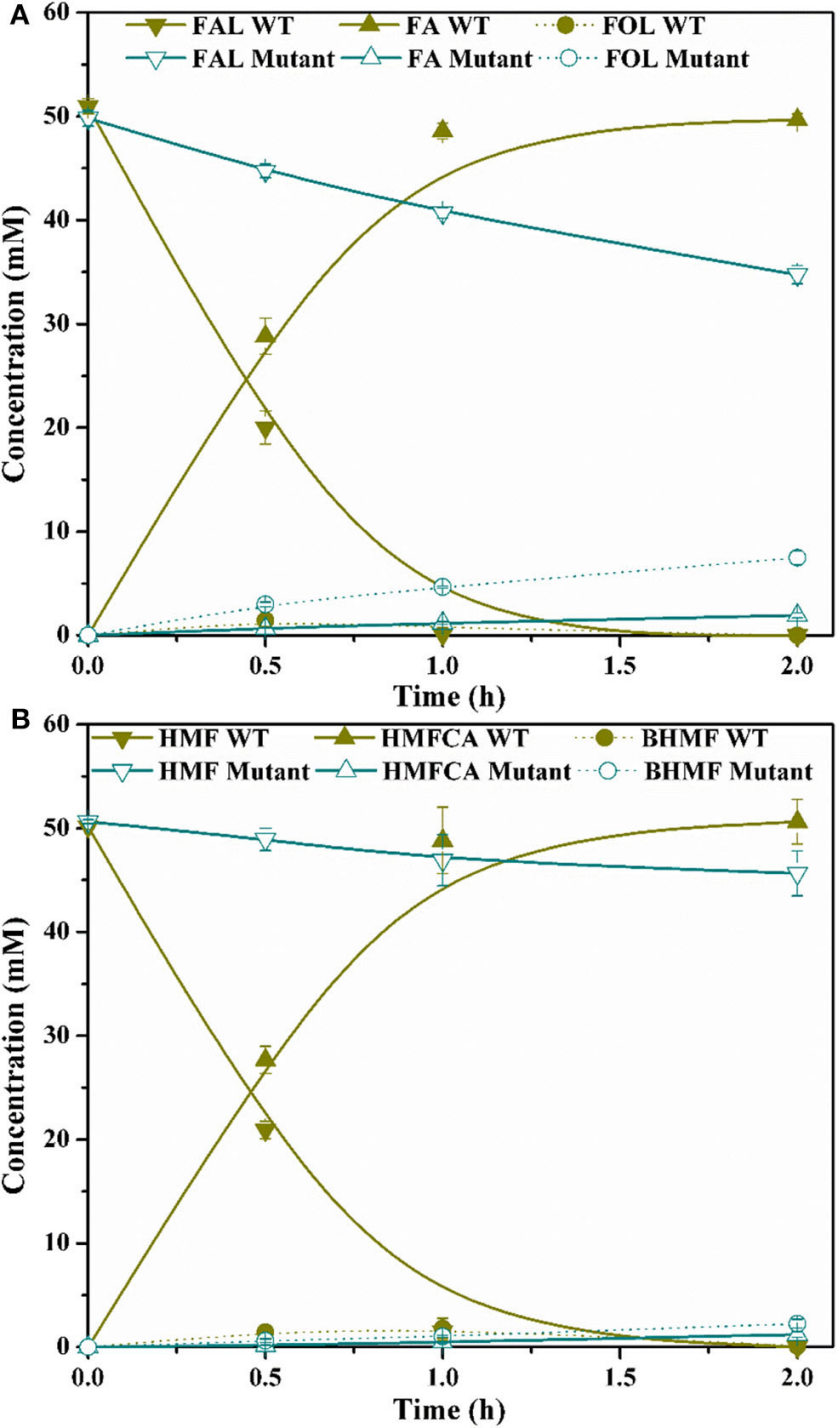
Time course of FAL conversion **(A)** and HMF conversion **(B)** by *P. putida* KT2440 wide-type (WT) and molybdate transporter mutant. Reaction conditions: phosphate buffer (200 mM, pH 6.0), 50 mM FAL **(A)** or HMF **(B)**, 10.5 g/L cells (dry weight), 25 mM CaCO_3_, 30°C, 200 rpm.

*G. oxydans* DSM 50049 and *C. testosteroni* SC1588 enabled similar selective oxidations as *P. putida* KT2440. Certain membrane bound enzyme was presumably suggested responsible for this reaction in *G. oxydans* DSM 50049 (Sayed et al., 2019), while several aldehyde dehydrogenase from *C. testosteroni* SC1588 were identified for the oxidation of furanic aldehydes into corresponding furancarboxylic acids in *E. coli* (Zhang X. Y. et al., 2020). In our research, based on genome annotation and sequence alignment, several candidate genes of *P. putida* KT2440 were selected and their functions in FAL oxidation were assessed through gene disruption. Regrettably, no substantial progress has been made up to now. During the inspection of the genome, a molybdate transporter (ModABC, PP_3828–PP_3830, Figure 6A) attracted our attention. As mentioned above, molybdate transporter had been evidenced critically in detoxification of many aromatic compounds (Blaschke et al., 1991; Ganas et al., 2008; Xia et al., 2018). In view of the facts that FAL and HMF were typical inhibitors to strains (Zaldivar et al., 1999; Franden et al., 2013), we thus wonder whether molybdate transporter was related to the biotransformation/detoxification of furanic aldehydes. We constructed a *modA*-disrupted mutant by homologous recombination (Figure 6B), which was routinely cultivated and prepared for FAL and HMF conversion. Figure 7 showed that the *modA*-disrupted mutant appeared to have lost the ability to oxidize substrates in a large extent. Although trace amounts of FA and HMFCA were detected, it was plausible that they were produced by the catalysis of certain unspecific aldehyde dehydrogenases. In addition, FOL and BHMF were evidently accumulated and couldn't be re-oxidized, which might be ascribed to the existence of excess furanic aldehydes. The key role of molybdate transporter suggested that one or more molybdoenzyme was involved in furanic aldehyde oxidation, which is essential for production of furancarboxylic acid. It is interesting to note that inactivation of molybdate transporter of *P. putida* KT2440 would substantially abolish its activity for vanillin oxidation (Graf and Altenbuchner, 2014), but it is still unclear whether these oxidation reactions were catalyzed by the same molybdoenzyme. Although molybdoenzyme had been proofed associated with aerobic degradation of FA in *P. putida* Fu1 and *C. basilensis* HMF14 (Koenig and Andreesen, 1989, 1990; Koopman et al., 2010), our study demonstrated its pivotal role in oxidation of FAL and HMF for the first time.

To effectively synthesize furan-based carboxylic acids *via* biocatalysis approach, an important challenge is to seek robust biocatalyst and characterize the functional enzyme(s). Presently, strains with strong ability to oxidize furanic aldehydes had been verified in several different species, covering *C. testosteroni, G. oxydans, N. coralline, Brevibacterium lutescens* etc. (Pérez et al., 2009; Zhang et al., 2017; Zhou et al., 2017; Sayed et al., 2019; Wen et al., 2020; Zhang R. Q. et al., 2020). On the contrary, there is a limited amount of researches aiming at identification and characterization of the relevant enzymes. The 3-succinoylsemialdehyde-pyridine dehydrogenase and vanillin dehydrogenase from *C. testosteroni* SC1588 enabled recombinant *E. coli* to oxidize FAL and HMF into FA and HMFCA, respectively (Shi et al., 2019; Zhang X. Y. et al., 2020), which was likely due to the wide substrate scope of 3-succinoylsemialdehyde-pyridine dehydrogenase and vanillin dehydrogenase instead of their main physiological functions *in vivo*. The enzyme responsible for furanic aldehydes oxidation seems intriguing to unravel both *in vivo* and *in vitro*. Identifying the molybdoenzyme involved in furanic aldehyde conversion in *P. putida* KT2440 was still ongoing in our laboratory, which would provide valuable enzyme resources for furan-based carboxylic acids production, as well as contribute to construction of recombinant strains with high furanic aldehydes tolerance.

## Conclusions

In summary, we have established an improved approach for selective synthesis of FA from FAL by *P. putida* KT2440. High FA titer was achieved in both batch conversion and fed-batch conversion, which not only addressed the challenge of FAL inhibition and toxicity, but also found an economically competitive process for FA production in large-scale. It is of interest to uncover the pivotal role of molybdate transporter in furanic aldehyde oxidation. The molybdoenzyme involved in the selective oxidation of formyl on furan ring can be expected to be developed as promising biocatalyst for the production of a series of furan-based carboxylic acids.

## Data Availability Statement

The original contributions presented in the study are included in the article/Supplementary Material, further inquiries can be directed to the corresponding author/s.

## Author Contributions

ZZ, QX, HT, and FZ conducted the experiments. QX and HT performed the whole-cell catalytic oxidation experiments. ZZ, QX, and FZ carried out gene knockout and analysis of the samples. ZZ wrote the manuscript and made substantial revisions. Supervision, funding acquisition, review, and editing of the manuscript were carried out by ZZ and JO. All authors have approved the final version of the manuscript.

## Conflict of Interest

The authors declare that the research was conducted in the absence of any commercial or financial relationships that could be construed as a potential conflict of interest.
