# Supplementary material for: Selective Biosynthesis of Furoic Acid From Furfural by Pseudomonas Putida and Identification of Molybdate Transporter Involvement in Furfural Oxidation
Source: Front Chem. 2020 Sep 25;8:587456. doi: 10.3389/fchem.2020.587456 (PMC7545826; doi:10.3389/fchem.2020.587456)
Supplement: Supplementary file 1 [file Presentation_1.pdf]

Supporting Information for

Selective biosynthesis of furoic acid from furfural by *Pseudomonas putida* and identification of molybdate transporter involvement in furfural oxidation

Running title: Biocatalytic synthesis of furoic acid

Zhaojuan Zheng[a,b*], Qianqian Xu[a], Huanghong Tan[a], Feng Zhou[a], Jia Ouyang[a,b*]

[a]*Jiangsu Co-Innovation Center of Efficient Processing and Utilization of Forest Resources, College of Chemical Engineering, Jiangsu Province Key Laboratory of Green Biomass-based Fuels and Chemicals, Nanjing Forestry University, Nanjing 210037, China*

[b]*Key Laboratory of Forestry Genetics & Biotechnology (Nanjing Forestry University), Ministry of Education, Nanjing 210037, China*

[*] Correspondence:

Zhaojuan Zheng. Tel: 86-025-85427587; Fax: 86-025-85427587; E-mail: zhengzj@njfu.edu.cn

Jia Ouyang. Tel: 86-025-85427129; Fax: 86-025-85427587; E-mail: hgouyj@njfu.edu.cn

Enzyme assay.

P. putida KT2440 was grown in LB medium at 30 ℃ and 200 rpm for 12 h on a rotary shaker. Then, 1% seed culture was inoculated to the fresh LB medium and cultivated under the same conditions. At the early exponential growth phase, 3 mM inducer (HMF, FAL and FOL, respectively) was added to the medium and cells were incubated for a total of 12 h. Thereafter, cells were harvested by centrifugation with 6,000 *g* for 8 min and washed twice with 0.85% NaCl saline solution. The cell pellets were re-suspended in phosphate buffer (50 mM, pH 7.0) and disrupted by sonication to obtain crude enzymes.

The activity of crude enzymes was assayed at 30 ℃ in 3 mL reaction mixture containing 50 mM phosphate buffer (pH 7.0), 5.0 mM FAL, 0.5 mM 3-(4,5-dimethylthiazol-2-yl)-2,5-diphenyltetrazolium bromide (MTT), 0.5 mM phenazine ethosulfate (PES), 1% (v/v) TritonX-100, and a suitable amount of crude enzymes. The absorbance change at 590 nm was measured for 1.0 min. One unit was defined as the amount of enzyme that reduced 1.0 μmol MTT per minute under the test condition.

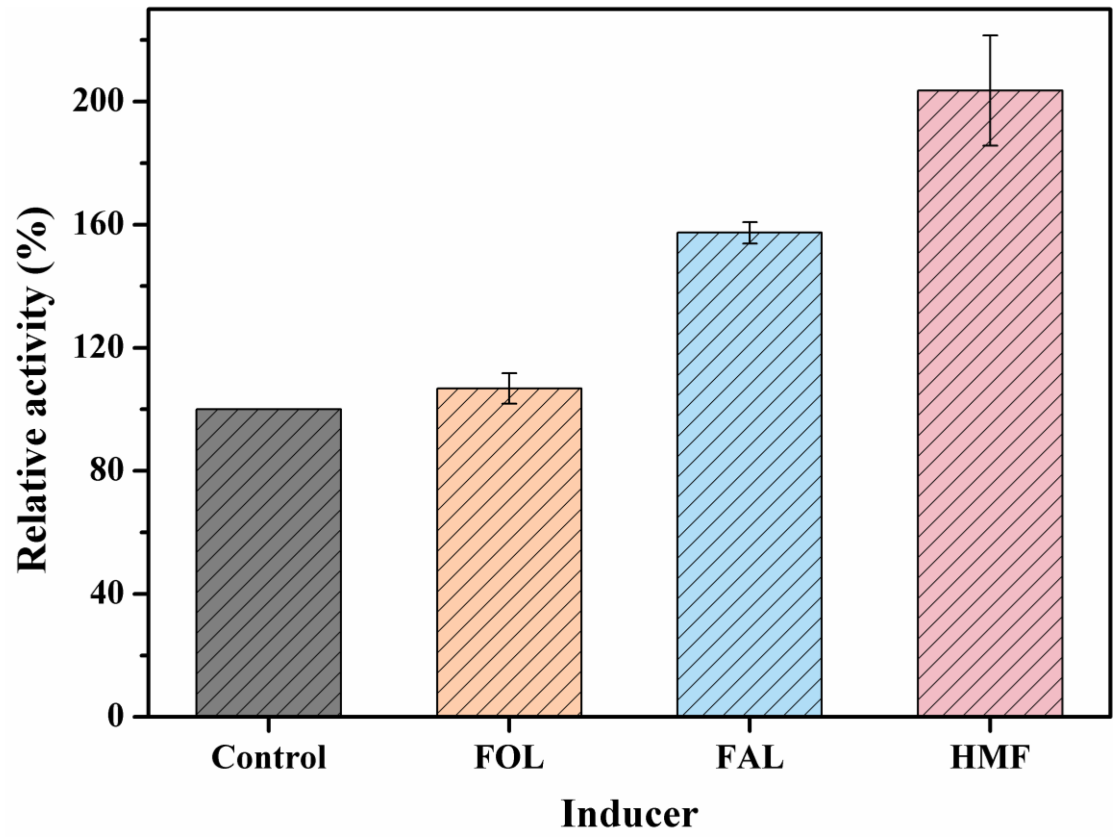

Fig. S1 Effect of different inducers on the activity of crude enzymes. All inducers were added with 3 mM at the early exponential growth phase, in accordance with Fig. 1a.

Table S1 Plasmids and oligonucleotide primers for *modA* disruption.

Name	Relevant characteristic
Plasmids	
pK18*mobsacB*	Allelic exchange vector, *ori*ColE1 Mob^+, *lacZα*, *sacB*; Km^r
pK18MS-Δ*modA*	The flanking regions of *modA* gene were inserted into pK18*mobsacB*
Primers	
*modA*up.f	GAATTCATGAGCGAGCTTGCACCG (*EcoR* I)
*modA*up.r	GCCGAGCCATGGGTTACCTTCACCGTTCTTGATCTGCGCG
*modA*down.f	CGCGCAGATCAAGAACGGTGAAGGTAACCCATGGCTCGGC
*modA*down.r	AAGCTTTTGCACCACGAACGGCATGG (*Hind* III)

Table S2 The recent work for whole-cell converting FAL into FA.

Biocatalyst	Reaction condition	FA titer	FA yield	FA selectivity	Reference
N. corallina B-276	Fed-batch, 12 h	92 mM	98.0%	–	Pérez et al., 2009
G. oxydans ATCC 621H	Fed-batch operation in a compressed oxygen supply-sealed and stirred tank reactor system, sodium hydroxide as neutralizer, 24 h	341 mM	91.1%	–	Zhou et al., 2017
Brevibacterium lutescens	Batch, corncob-derived FAL, 18 h	103.4 mM	100%	–	Zhang et al., 2020
Recombinant *E. coli* harboring CtSAPDH[a]	Fed-batch, $CaCO_3$ as neutralizer, 96 h	147 mM	100%	100%	Shi et al., 2019
	Fed-batch, $NaHCO_3$ as neutralizer, 96 h	129 mM	–	84.3%	
Recombinant *E. coli* harboring HLADH[b]	Batch, FAL or rice straw-derived FAL, 96 h	75 mM	99.9%	100%	Peng et al., 2019
Recombinant *E. coli* harboring HMFO[c]	Batch, FAL, 96 h	50 mM	100%	100%	Wang et al., 2020
P. putida KT2440	Batch, $CaCO_3$ as neutralizer, 2 h	170 mM	97.1%	100%	This study
	Fed-batch, $CaCO_3$ as neutralizer, 3 h	204 mM	97.5%	>97%	

[a]CtSAPDH, 3-succinoylsemialdehyde-pyridine dehydrogenase from *C. testosteroni* SC1588.

[b]HLADH, horse liver alcohol dehydrogenase.

[c]HMFO, HMF oxidase from *Methylovorus* sp. MP688.

Reference

Peng, B., Ma, C.L., Zhang, P.Q., Wu, C.Q., Wang, Z.W., Li, A.T., et al. (2019). An effective hybrid strategy for converting rice straw to furoic acid by tandem catalysis *via* Sn-sepiolite combined with recombinant *E. coli* whole cells harboring horse liver alcohol dehydrogenase. *Green Chem.* 21, 5914−5923.

Pérez, H.I., Manjarrez, N., Solís, A., Luna, H., Ramírex, M.A., Cassani, J. (2009). Microbial biocatalytic preparation of 2-furoic acid by oxidation of 2-furfuryl alcohol and 2-furanaldehyde with *Nocardia corallina*. *Afric. J. Biotechnol.* 8, 2279–2282.

Shi, S.S., Zhang, X.Y., Zong, M.H., Wang, C.F., Li, N. (2019). Selective synthesis of 2-furoic acid and 5-hydroxymethyl-2-furancarboxylic acid from bio-based furans by recombinant *Escherichia coli* cells. *Mol. Catal.* 469, 68−74.

Wang, Z.W., Gong, C.J., He, Y.C. (2020). Improved biosynthesis of 5-hydroxymethyl-2-furancarboxylic acid and furoic acid from biomass-derived furans with high substrate tolerance of recombinant *Escherichia coli* HMFOMUT whole-cells. *Bioresour. Technol.* 303, 122930.

Zhang, R.Q., Ma, C.L., Shen, Y.F., Sun, J.F., Jiang, K., Jiang, Z.B., et al. (2020). Enhanced biosynthesis of furoic acid via the effective pretreatment of corncob into furfural in the biphasic media. *Catal. Lett.* 150, 2220–2227.

Zhou, X., Zhou, X., Xu, Y., Chen, R.R. (2017). *Gluconobacter oxydans* (ATCC 621H) catalyzed oxidation of furfural for detoxification of furfural and bioproduction of furoic acid. *J. Chem. Technol. Biot.* 92, 1285−1289.
